# DNA-Based Tools to Certify Authenticity of Rice Varieties—An Overview

**DOI:** 10.3390/foods11030258

**Published:** 2022-01-19

**Authors:** Maria Beatriz Vieira, Maria V. Faustino, Tiago F. Lourenço, M. Margarida Oliveira

**Affiliations:** Instituto de Tecnologia Química e Biológica António Xavier (ITQB NOVA), Universidade Nova de Lisboa Av. da República, 2780-157 Oeiras, Portugal; marybtv@itqb.unl.pt (M.B.V.); mariafaustino@itqb.unl.pt (M.V.F.); tsantos@itqb.unl.pt (T.F.L.)

**Keywords:** adulteration, DNA barcoding, DNA-markers, fraud, high-resolution melting, isothermal amplification, multiplex, PCR, SNPs, SSRs

## Abstract

Rice (*Oryza sativa* L.) is one of the most cultivated and consumed crops worldwide. It is mainly produced in Asia but, due to its large genetic pool, it has expanded to several ecosystems, latitudes and climatic conditions. Europe is a rice producing region, especially in the Mediterranean countries, that grow mostly typical *japonica* varieties. The European consumer interest in rice has increased over the last decades towards more exotic types, often more expensive (e.g., aromatic rice) and Europe is a net importer of this commodity. This has increased food fraud opportunities in the rice supply chain, which may deliver mixtures with lower quality rice, a problem that is now global. The development of tools to clearly identify undesirable mixtures thus became urgent. Among the various tools available, DNA-based markers are considered particularly reliable and stable for discrimination of rice varieties. This review covers aspects ranging from rice diversity and fraud issues to the DNA-based methods used to distinguish varieties and detect unwanted mixtures. Although not exhaustive, the review covers the diversity of strategies and ongoing improvements already tested, highlighting important advantages and disadvantages in terms of costs, reliability, labor-effort and potential scalability for routine fraud detection.

## 1. Introduction

### 1.1. Rice Diversity

Rice is a major crop that directly feeds about half of the global population, most of it in developing countries [1]. Following a tendency for human population growth (12% from 2010 to 2020), global rice production has also risen in the same period, reaching 500 million tons (13% increase) [2]. This was possible due to improved agronomic practices and improved varieties.

Rice currently grows over a wide range of ecosystems, and is continuously targeted for varietal improvement and for evaluation and preservation of germplasm collections. Numerous researchers and institutions have been contributing to bringing forgotten varieties into cultivation, as well as to increase rice diversity, thus adding to its natural genetic spectrum. Diversity is crucial for breeders, as a source of genetic traits to introgress into commercial varieties, either for pest and disease resistance, abiotic stress tolerance, grain nutritional quality, or others. Sometimes, crosses are even made with distantly-related genotypes, as a way to introduce specific new traits. Due to all of this activity, and despite having been one of the first domesticated species, cultivated rice still explores a very broad gene pool.

There are more than 500,000 rice accessions in gene banks worldwide [3]. To better explore the molecular diversity of this germplasm, a large sequencing initiative, the 3K Rice Genome Project [4], was launched by the Chinese Academy of Agricultural Sciences, BGI-Schenzhen and the International Rice Research Institute (IRRI). The 3K-RG project targeted the most relevant rice species, *Oryza sativa*, and re-sequenced 3000 rice genomes selected from the available diversity, generating an unprecedented resource for large-scale discovery of genetic variation in this species [5]. 

*O. sativa* originated in Asia and is now cultivated all over the world. However, *Oryza glaberrima*, originally from West Africa, has also been domesticated and commercialized, although at a very reduced scale, and nowadays it only shows up in a few African markets. Furthermore, 24 wild species found in Asia, Africa, Australia and the Americas, and nine related genera have also been identified and are now part of the IRRI International Rice Genebank [6].

Genetic structure studies of *O. sativa* accessions clearly indicate two major subgroups, *indica* and *japonica*, likely originated from independent domestication events. Two other ones of less clear origin (*aus-boro* and *sadri-basmati*), and a fifth group that different authors consider either as derived from the *japonica* subdivision into temperate and tropical types, or as an admixtures group [7,8,9] have also been identified. The data analyses of the 3000 rice sequenced genomes were consistent with five major groups, but additional subpopulations were identified suggesting nine subpopulations correlated with geographic origin [1].

Recently, the integration of geographic, environmental, archaeobotanical and paleoclimate data with whole-genome sequencing of more than 1400 rice landraces, revealed that *O. sativa* originated at the Yangtse valley, about 9000 years ago, and then diversified into temperate and tropical rice [10]. This evolution happened during a global cooling episode, about 4200 years ago, allowing rice to reach Southeast Asia (2500 years ago) and rapidly diversify thereafter [10]. 

**Figure 1:**
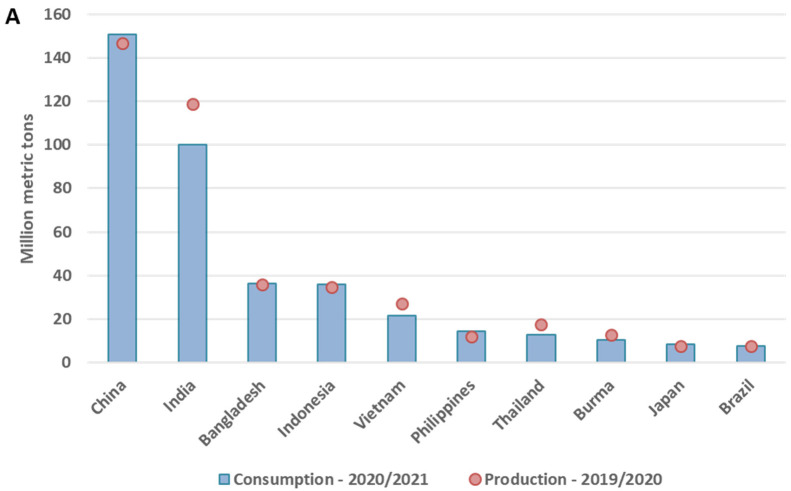

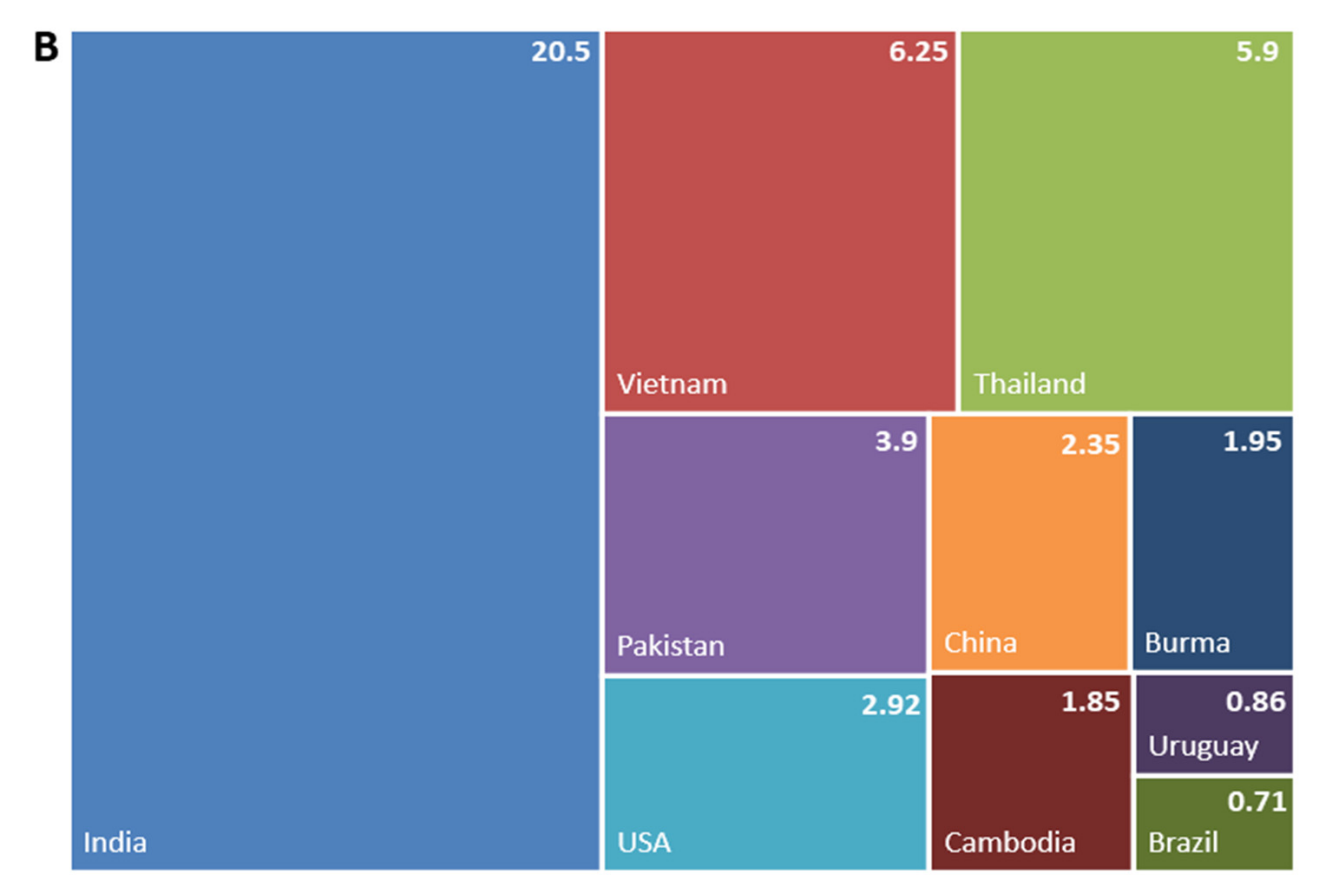
Identification of the 10 larger consuming, producing and exporting countries of the world (**A**) Production of milled rice in 2019/2020, and rice consumption in 2020/2021 in each of the 10 top producing/consuming countries. (**B**) The 10 main rice exporting countries worldwide with values of exportation in million metric tons. (Graphs were built based on data in [2]).

Nowadays rice is being produced in all five continents, but more significantly in Asia, in particular in India and China, where consumption is also higher (Figure 1A). India, Vietnam and Thailand are producing more than they consume, and they are also the most significant exporters, followed by Pakistan and USA (Figure 1B).

### 1.2. Rice in Europe and the Mediterranean Region—Production and Market

Rice history in Europe is relatively recent, and it was probably only around the 15th century that it became an established crop in this region [9,11]. In Europe, rice production has slightly declined in recent years, from average 4.1 million tons/year in the period of 2013–2016 to 3.9 million tons/year (estimated in 2017–18). Several environmental and socio-economic constraints justified such reduction, which was also observed in North America and Oceania [12].

Rice currently grows in eight European Union countries (Bulgaria, France, Greece, Hungary, Italy, Portugal, Romania and Spain) and its cultivation occupies approximately 460,000 ha in EU countries with a total production of approx. 2.8 million tons in 2017 (1.7 million tons, milled basis) [12]. In 2020, the EU produced 5.6 million tons of paddy rice, corresponding to 3.358 million tons of whole grain (milled rice equivalent) [13].

Outside the EU, but still in the Mediterranean area, Turkey and Egypt are the most significant countries for rice production, with 789,000 ha of cultivated area. In 2005, Egypt produced 6.2 million tons in an area of approximately 652,000 ha. Egypt’s production has slightly increased in the past decade, almost reaching 6.7 million tons in 2019 [14,15]. The European rice market is mainly sub-divided in two segments, *japonica* (short/medium grain), and *indica* (long grain, accounting for about 25% of consumption). In terms of production, the so-called *indica* rice grown in Europe is not real *indica*, and in the frame of this review we call it *indica*-type rice. This *indica*-type rice has some *indica* grain features but mostly a temperate *japonica* genetic background. European rice genetic background was characterized by Courtois et al. [9,16] in the frame of a European project focusing on the European Rice Germplasm Collection. The authors targeted 425 accessions (mostly from Italy, Spain, Greece, Portugal and France, with several from Bulgaria, Hungary, Romania, Turkey and Russia), comparing them with a reference set of 50 accessions representing *O. sativa* diversity. The accessions originated mostly from temperate areas in Asia and America, but many resulted from European breeding programs. This pedigree study explored Single Nucleotide Polymorphisms (see Section 3.1.5) and revealed that European rice is mostly temperate japonica. The few non-japonica accessions were mainly introductions from South Asia and, in most cases, brought to Europe as potential donors. Only two accessions (derived from European breeding programs) were classified as *indica*. Backcrossing to the recurrent *japonica* parent is usually needed to recover fertility and for adaptation to local climatic conditions. This was actually the strategy used to introgress the semi-dwarfing gene (*sd1*) into traditional Portuguese varieties [17].

The agronomically-relevant traits often targeted by breeders are unknown to the consumer, who concentrate more on characteristics such as size, shape, color, fragrance or cooking properties of the grains. Equally attracted by exotic rice, the EU consumer appreciates *indica* and *aromatic* varieties, which makes the region a net importer of this rice. The increase in rice consumption in the EU, allied to a slightly reduced rice production, is increasing the external dependence on this commodity. 

EU is already importing about one-third of its internal consumption [18]. Most rice imported to EU comes from Pakistan, India, Thailand, Cambodia, Myanmar and Guyana (listed by decreasing relevance in terms of imported volumes). Although *indica* rice is almost always more expensive, an increase in market prices for *japonica* rice (together with a decrease in the *indica* price) has also contributed to increase *indica* importations [19]. Although EU produces *japonica* rice, the increase in its price has led the market to search for *japonica* rice from third countries. This increase in EU importations of *japonica* rice, has led to some of the biggest consumer countries to come together to promote EU *japonica* rice production and sales in the European market [20]. 

### 1.3. The Problem: Fraud in Trade Varieties

There are numerous rice varieties being cultivated and commercialized all over the world. The increasing rice demand in Europe, and rising dependence on rice produced elsewhere is, therefore, increasing the risk of adulteration and fraud. This tendency is a result of globalization and a growing problem in the food sector in general. Fraud is profit-motivated and usually targets the most expensive products substituting them by lower quality, cheaper ones. However, when large volumes are involved, as it happens for commodities such as rice (or milk), these also become fraud targets. 

The supply chains, from production to consumption, are attacked wherever the vulnerability is higher, and significant problems have been reported in a number of sectors and value chains. In the food industry, fraud business is estimated to reach 30 billion USD profit per year [21]. When it compromises food safety, fraud may also constitute a health issue. Food fraud was recently analyzed by FAO for the region of Asia and Pacific, where globalization and high demand for premium quality products have significantly augmented the problem [22]. To address this issue, the Europol INTERPOL operations (known as OPSON) have been active since 2011 to target fake and sub-standard food and beverages circulating throughout the global economy [23]. The SARS-CoV2 pandemic has affected surveillance schemes on some food products, probably contributing to increased fraud, but the extension of the problem can only be fully assessed when normal scrutiny is back in place. The disruption of food supply chains as a result of the pandemic has increased food cost and, as consequence, in 2020, the rice price reached its highest level in recent years, and it increased further in the first quarter of 2021, declining thereafter [19]. 

**Figure 2:**
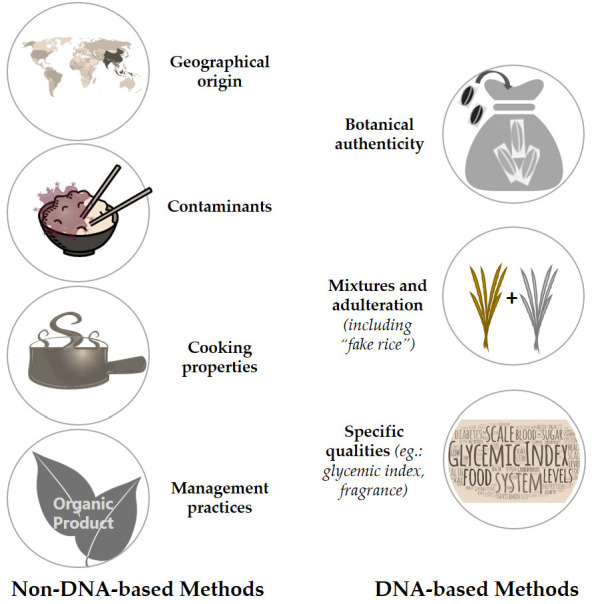
Schematic representation of some of the most common types of rice fraud and the preferred type of strategy (DNA or non-DNA-based) for their detection. Whenever the rice has features that result from the interaction of plant genetics and the environment (including agricultural practices, rice processing, long-term or inadequate storage, contamination with toxic compounds, pollutants etc.), DNA-based techniques are not appropriate to detect fraud, and other methods must be employed.

Concerns about rice fraud vary substantially depending on the region. The substitution of high-quality by low-quality rice is usually the main concern, but other relevant issues include the identification of region of origin, or specific qualities such as low glycemic index or other certified traits (such as aroma) are other relevant issues (Figure 2). A good revision of fraud in global rice trade has been made by Śliwińska-Bartel et al. [24], who also cover several issues, detection strategies and scientific studies targeting rice botanical origin (of Basmati, Italian, Jasmine and other rice), geographic origin (Asia, South America and other continents) and even cultivation methods (e.g., organic rice).

To address the different types of concerns, several methods have been developed and applied in various contexts. These have been increasing in sensitivity and are often based on the knowledge of rice grain morphology, (bio)chemical composition (including minerals, proteins, carbohydrates, lipids, vitamins and volatile compounds) and cooking properties. DNA-based strategies to detect potential fraud have also been employed on rice (as well as on many other food products) using targeted or untargeted screening aiming at varietal discrimination. These, however, are not appropriate for the detection of all possible types of fraud. In Figure 2 we schematically present the main types of fraud highlighting those in which DNA-based strategies may be helpful. 

In Section 2 we briefly cover the non-DNA based methods currently employed on rice, and in Section 3 we focus on DNA-based strategies with several sub-sections covering not only the more traditional methodologies, but also recent advances. Finally, in Section 4 we briefly present the main conclusions, summarizing in a comprehensive table the DNA-based techniques used for rice varietal certification.

## 2. Brief Overview of Non-DNA Based Methods for Rice Certification

Traditionally, morphological properties were used as criteria for the differentiation of rice varieties and groups [25,26]. To examine the morphology of the kernel, techniques such as electron microscopy and image analysis were employed [26,27,28,29]. Authenticity of rice products were also verified through differences in the amylose content, protein, flavor and texture attributes [18,22]. These characteristics were able to broadly divide rice into main groups, but they lack the ability to differentiate cultivars, while also being expensive and inefficient for application in commercial settings [26,27]. 

The detection of specific proteins was widely used to reveal adulterants, involving electrophoretic and immunological methods such as SDS-PAGE and immunoassays [30]. Although useful to detect adulterants, protein-based methods can mainly group cultivars and are less appropriate to discriminate between them [26,27].

Recently, new analytical and spectroscopic techniques have been increasingly used as alternative technologies for the authentication of rice varieties based on both botanical and geographic characteristics [31]. Analytical methods are mostly based on separation by gas chromatography or liquid chromatography and analysis by mass spectrometry, or on inductively coupled plasma-mass spectrometry (ICP-MS) [32]. These methods were successfully employed to identify the geographic origin of different types of rice [33,34,35] and they proved particularly useful for the detection of contaminants and adulterants [36,37,38].

Spectroscopic techniques are emerging as alternatives for rice authentication. These include laser-induced breakdown spectroscopy, near-infrared spectroscopy, nuclear magnetic resonance spectroscopy and Raman spectroscopy [32]. These methods were successfully employed for both rice authentication [39,40,41,42] and detection of chemical contaminants and adulterants [43,44,45,46]. 

Analytical methods can be highly efficient and reliable in the identification of the geographical origin and presence of adulterants, however they exhibit strong limitations in the detection of contaminant varieties [24]. To overcome these limitations, DNA-based methods have been selected as a source of information providing strong reliability, while also being less prone to alterations resulting from rice processing.

**Figure 3:**
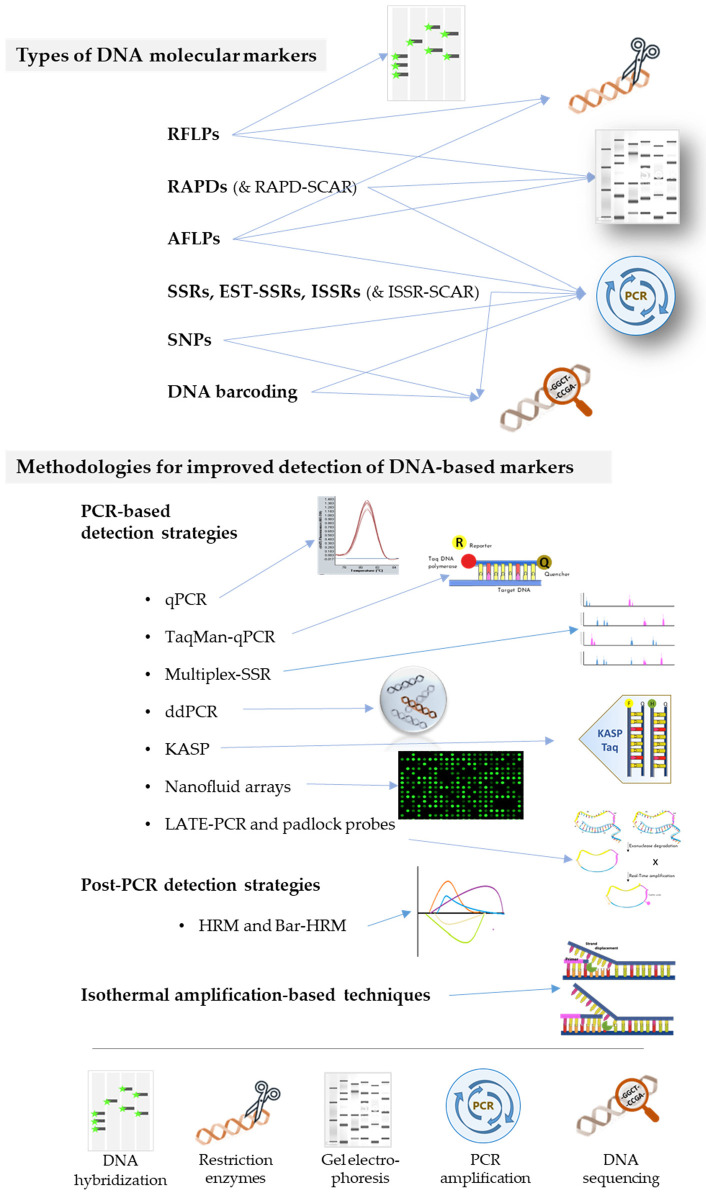
Schematic representation of the most common types of DNA markers used in rice authentication, indicating the key methods that characterize each one (legend in the bottom), and some of the methodologies used to improve marker detection.

In Figure 3 we briefly summarize some key features of these methodologies.

## 3. DNA-Based Tools for Rice Varietal Certification 

Molecular DNA-based strategies have been widely explored and used in authentication and traceability of various food-based products along the industrial processing (reviewed by Fanelli et al. [47]). Most DNA-based approaches rely on the amplification of target molecular DNA-based markers through Polymerase Chain Reaction (PCR), offering high specificity and sensitivity, enabling the discrimination among genotypes [48]. The application of these molecular approaches in rice industry, for verifying variety labelling and composition in seed lots, was boosted by the availability of whole genome sequences of both *indica* and *japonica* cultivars and proved efficient, especially for purity assessment of aromatic cultivars [27,49]. The use of well-defined molecular markers, and recent improvements in PCR-based strategies paved the ground for the development of new, more automated tools for tackling adulteration and fraud in the rice industry, as briefly covered below and schematically represented in Figure 3.

### 3.1. Types of DNA Molecular Markers

Molecular markers can be defined as regions of the DNA that are easily detected and quantified in a population. They consist of natural occurring polymorphisms and are usually associated with inheritance of specific genes/traits of interest. These marker regions are naturally generated by different types of mutations that occur throughout the genome, particularly in non-coding regions, where they are the more abundant, stable and less sensitive to environmental influence [50].

Such markers have been widely used in rice genotyping and marker-assisted breeding, as well as in rice authentication and fraud detection [51,52,53,54,55]. Most of these markers, can be easily detected by PCR, which is considered the easiest and most inexpensive tool for molecular authentication of food products [56] providing both qualitative and quantitative data [57,58,59,60]. Rice has been characterized by a number of different types of markers, including RFLPs, RAPDs, AFLPs, SSRs and SNPs (and some variations or specifications to these techniques, such as SCARs, EST-SSRs, ISSRs, InDels) as described in the following sub-sections.

#### 3.1.1. Restriction Fragment Length Polymorphisms (RFLPs)

RFLP (Restriction Fragment Length Polymorphism) explores the use of restriction enzymes to generate fragments that are separated by electrophoresis and a target fragment is revealed through hybridization with a labelled probe [61,62]. Markers are generated by changes in fragment size due to (a) nucleotide(s) insertion or deletion and (b) variations in the target restriction sites. RFLPs were largely used in rice to search for intra-specific genetic variability and mapping research [63]. Interestingly, a patent was recently released providing a simple and efficient PCR-RFLP marker able to discriminate rice varieties based on aroma [64]. This method is able to identify adulteration of aromatic samples with non-aromatic rice [64]. Nonetheless, RFLPs are an expensive and labor consuming strategy, requiring high-quality DNA and labelled probes, as well as having a very limited capacity for scaling up, thus far from ideal for routine fraud detection [56,65].

#### 3.1.2. Random Amplification of Polymorphic DNAs (RAPDs)

The Random Amplification of Polymorphic DNA (RAPD) strategy has also been employed for rice discrimination. It is based on PCR, uses low amounts of DNA with no need for high purity, and does not require previous knowledge of the host genome. In spite of these advantages, RAPD does not offer enough reliability since it uses short primers (of about 10 nucleotides) and low annealing temperatures to amplify random regions in the genome, which makes the strategy hardly transferable across laboratories [62,66]. Still, RAPDs have been employed to discriminate aromatic rice varieties [67,68]. To increase reliability in the distinction of aromatic from jasmine rice, Wu et al. [54,67] have identified RAPD fragments using primer pairs that were specifically designed for pairing to a sequence characterized amplified region (SCAR). SCAR primers can only be designed after sequencing a selected fragment identified from RAPD, thus allowing more restrictive annealing temperatures in PCR analyses and thus, higher reliability. Nevertheless, these markers are still not adequate for detection of rice mixtures or mislabelings.

#### 3.1.3. Amplified Fragment Length Polymorphism (AFLPs)

Amplified Fragment Length Polymorphism (AFLP) has been used to study rice biodiversity [69] and could also be considered to detect mislabeling. AFLP uses restriction enzymes recognizing frequent and rare restriction sites in the genome to generate fragments (that end with frequent or rare sticky ends, or a combination of both), some of which are later specifically selected for PCR amplification. The selection is achieved with primers made of double-stranded adaptors linked to sequences complementing those generated by the restriction enzymes, and additionally carrying 1 to 3 nucleotides, to reduce the number of fragments for amplification. For higher specificity, this is usually completed in two steps, with a pre-amplification using only 1 nucleotide and a final amplification using 2 to 3 selective nucleotides. AFLPs can efficiently reveal multiple polymorphisms in a single reaction, however, they require a high amount of high quality DNA, and a very good electrophoretic separation to reduce chances of having a single band including more than one amplification product [56,70,71,72,73,74]. Due to these characteristics, AFLPs are not considered ideal for rice authenticity evaluation.

#### 3.1.4. Simple Sequence Repeats (SSRs)

The most used molecular markers for rice traceability studies are Simple Sequence Repeats (SSRs) (Table 1), also known as microsatellites [27]. These are sequences of 1 to 6 nucleotides in tandem repeats of 20 bp or more (Class I) or of 12–19 bp (Class II). SSR polymorphism relies on the number of repeats present in a target DNA region. They can be detected by PCR using primers for the conserved regions that flank them. SSRs are present in coding and non-coding regions of all 12 rice chromosomes as well as in organellar DNA. Studies have shown that their distribution is not random and location can identify specific genes [75,76].

These markers have been the most applied strategy to tackle adulteration and traceability in rice industry, and for the assessment of rice seeds genetic purity (Table 1) [24,27,77]. Rice SSRs of class II were found hypervariable, but those of Class I were found to be more polymorphic [78,79] and a powerful tool for correlating genetic maps with genomic sequences [79]. SSRs have been reported in the identification and quantification of adulteration of traditional basmati rice (with non-basmati varieties or non-premium long-grain rice), mainly through the use of fragrance-related markers [49,57,77,80,81,82]. Microsatellites have also been targeted in multiple combinations in several PCR-based systems, being detected using a variety of separation and detection methods to achieve optimal discrimination capacity [57,77,83]. SSRs have also been applied in direct evaluation of rice seed lots [53,84], which may allow automation and thus reduce the necessary person-power required.

A specific use of SSRs, known as EST-SSRs (Expressed Sequence Tag derived SSRs), explores the targeting of coding regions of the genome by using fragments of cDNA (DNA reversely transcribed from messenger RNA expressed in specific developmental stages or conditions) as templates to screen for repetitive sequences. This technology has been receiving special attention, since the polymorphisms obtained may be directly related to biological functions and phenotypes. EST-SSRs are thus valuable markers for purity evaluation of seed lots and varietal discrimination [75,85,86]. A less used variation to SSRs that has also been applied for rice genetic fingerprinting, targets inversely oriented regions between adjacent SSRs and is called ISSR (Inter-Simple Sequence Repeat). ISSRs have also been used to discriminate rice varieties [87,88] and, when combined with SCAR markers (in this case, SCAR markers make use of the sequence information of the ISSR amplified locus to specifically amplify it by PCR), they could precisely identify a Chinese glutinous rice [89].

#### 3.1.5. Single Nucleotide Polymorphisms (SNPs)

Single base variations in the DNA sequence, defined as single nucleotide polymorphisms (SNPs), are relevant DNA markers. Actually, they constitute the most abundant type of marker present in the rice genome [90] and, opposite to other markers, their identification does not require DNA separation by size. Their biallelic nature allows for simpler detection and quantification of allelic variation [70]. SNPs can be identified from overlapping sequences obtained from genome or EST sequencing, or from other DNA-fingerprinting strategies. However, their use depends on appropriate SNPs cataloguing and on the availability of genotype-resequencing data [91]. The advances in Next-Generation Sequencing (NGS) technologies, together with improved bioinformatic tools, have boosted the annotation of SNPs along the genome [47,92]. In rice, the 3K-RG re-sequencing project was vital for the identification and annotation of SNPs. From this project, 29 million SNPs were identified and annotated [93,94], which was critical for SNP widespread use for varietal genotyping certification, using, for instance, genotype-by-sequencing (GBS) strategies.

Another relevant tool is the Rice SNP-Seek database [95,96] that includes the phenotypic, genotypic and varietal information of rice and SNP genotyping data from the 3K Rice Genome project. Ultimately, this paved the way for the identification of millions of SNPs and InDels that assisted in rice genomics research. These genetic markers are essential for the differentiation of rice genotypes and may support the development of DNA-based rice authentication techniques [1,51,97].

SNPs application to variety identification can be performed by systems detecting a single marker at a time by PCR-based methods [98,99]. This is, however, expensive and time-consuming. High-throughput approaches based on the exploitation of multiple markers in a single reaction (using optimized primers and information on SNPs localization) can change this scenario, providing an affordable and time-efficient alternative for varietal identification [100,101]. Currently, there are various highly automated, efficient, and relatively inexpensive methods, including direct DNA sequencing or denaturing high performance liquid chromatography (dHPLC). However, the TaqMan assay that we discuss in Section 3.2.1 is highlighted as particularly convenient for SNP detection. 

#### 3.1.6. DNA Barcoding

DNA barcoding explores a unique pattern of variation in a DNA section or gene that allows the specific identification of a species or variety, and it was already proposed as a standardized method [102]. This technique is deeply dependent on DNA amplification and sequencing, and analyses of orthologous DNA regions for species identification [102]. Nonetheless, the search for a DNA region that has low variability within a *taxon*, but high interspecies variability may represent a major challenge in some cases. Interestingly, in plants, DNA barcoding depends mainly on chloroplast genome sequence data. The use of the chloroplast genome has several advantages, (1) it has a simple and stable genetic structure, (2) it is haploid and recombination is rare and (3) it is generally uniparentally transferred [103]. Indeed, plastid DNA barcoding was tested to discriminate among *Oryza* species [103]. The authors concluded that a better standardization of universal primers is needed to improve amplification efficiency and detect polymorphisms [103]. Recently Zang et al., 2021 [104] explored DNA barcoding to discriminate 21 species of *Oryza*. In this case, the authors compared the performance of conventional plant DNA barcodes with rice-specific chloroplast and nuclear DNA barcodes, and a chloroplast genome super DNA barcode. Chloroplast genome super DNA barcodes uses the complete genome or a part of it that contains enough amount of information to allow discriminating genotypes. In this study the super barcode was the whole chloroplast genome, and it actually proved to be the most reliable marker, although it required extensive sequencing and informatic analyses. A universal plant DNA barcoding region is within the *trnL*-F region of the chloroplast genome.

As mentioned before, a crucial component of DNA barcoding is the availability of high-quality sequencing data. A major step towards the advancement of DNA barcoding technology in rice was the 3K Rice Genome Project (3K-RG) initiative [4], since it allowed for the identification and exploration of allelic/haplotype variation [105]. From the 3K-RG project, several databases arose such as RPAN genome browser and the already-mentioned Rice SNP-Seek database [95,96]. RPAN genome browser represents the union of all the genes present in rice species, providing a new dimension to the genome complexity based on the presence or absence of variation in a genome [106]. 

With the decrease in whole genome sequencing price and, consequently, the increase in the number of available sequenced genomes, the number of DNA barcodes is rising thus providing new valuable information to be employed to detect fraud worldwide [107].

### 3.2. Methodologies for Improved Detection of DNA-Based Markers for Rice Authentication

The development of a wide variety of molecular methods, over the last years, has boosted PCR-based techniques improving their accuracy, reliability, speed and automation, in the detection of DNA markers. Thermocyclers (the equipment needed for PCR amplifications) are nowadays available in all molecular biology laboratories. Along with the low cost equipment and reagents, PCR requires a small amount of template DNA and can analyze multiple markers, simultaneously in a single sample [49]. Nonetheless, other techniques involving fragment amplification without a thermocycler, may also prove worthy in specific cases, as briefly explained in Section 3.3.

The detection technologies described below have been used in both the detection of food adulterants and in the discrimination of rice varieties.

#### 3.2.1. PCR-Based Detection Strategies (qPCR, TaqMan-qPCR, Multiplex-SSR, ddPCR, KASP, Nanofluid Arrays, LATE-PCR and Padlock Probes)

Developed over two decades ago, Real-Time PCR, also known as Quantitative PCR (qPCR), rapidly emerged as an important tool to detect contaminants and adulteration in the food industry. Real-time PCR has several advantages, when compared with classical PCR, namely its higher sensitivity and specificity, also allowing the simultaneous processing of multiple samples with short hands-on time, and the automated, simple and reproducible detection of the amplified products without need for gel electrophoresis for product visualization [108,109]. To explore the qPCR potential, several modifications were introduced including TaqMan-based Real-time PCR, multiplex-SSR, droplet digital PCR, Kompetitive Allele PCR, nanofluid arrays, and padlock probe ligation with multiplex microarray detection, among others. 

TaqMan-based Real-time PCR is based on the use of fluorophore-labelled nucleic acid probes complementary to the target DNA, and explores the exonuclease activity of Taq polymerase to release the fluorophore that is then quantified. Although expensive, the technique is very accurate with 1% detection limit [110]. This technology has been used in the authentication of animal feeds [108] and it also allowed the identification of economically important adulteration of Basmati rice [60].

Another variation of Real-time PCR, is the multiplex microsatellite marker assay (multiplex-SSR). This method involves a single-tube assay where a panel of microsatellite loci are used to generate variety-specific allele profiles. Using a panel of eight microsatellite loci, it was possible to detect up to 1% adulteration in Basmati rice samples [57]. Similarly, a duplex real time PCR method was developed to unravel adulteration in basmati samples (“NoBa”) [58]. This method targeted a region of the DNA that contains seven bases deleted in Basmati vs. non-Basmati. The NoBa method proved to have a good correlation with the SSR-based method described above [58].

A recently developed assay for absolute quantification of DNA copy number is droplet digital PCR (ddPCR). This technique was already employed in rice authentication [111] since it overcomes some of the limitations of quantitative Real-time PCR [112]. In ddPCR, the reactions are distributed in 20,000 droplets, the expression data is only collected at the end of the PCR, being then statistically analyzed and providing robust data [113] that translate into direct quantification (without standard curve) and higher accuracy. Since the fluorescence is measured at the end of the reaction, the expression quantification is independent of PCR efficiency [113]. This method was applied to uncover adulteration in Basmati rice samples, showing potential to quantify non-Basmati content of up to 1% [111]. 

Recently, the KASP technology, based on Kompetitive Allele PCR, has been applied in *Oryza* species and subspecies discrimination, for identification of commercial basmati rice varieties using SNP and InDel markers [98,114]. KASP technology is based on the use of primer sequences that are allele-specific and in the use of fluorophore probes to detect DNA targets that are different in a single nucleotide. KASP detects biallelic SNP polymorphism [47,98,114]. Another SNP genotyping system uses nanofluid arrays. This method relies on the use of integrated fluidic circuits, allowing the simultaneous analysis of multiple samples (up to 96 samples). This method has been used for variety discrimination in various agri-foods [47] and its application in genotyping rice sub-species was recently explored [101].

A system previously developed to identify non-authorized GMOs, based on padlock probe ligation and a multiplex microarray detection [115], was also tested for rice certification. This method relies on amplification by Linear-After-The-Exponential (LATE)-PCR, which is an asymmetrical PCR that generates single-stranded sequences. The method was tested to identify Basmati presence in non-fragrant rice, and it was considered an efficient and reliable tool for detecting small amounts of DNA in mixtures [116].

The current technological advances are constantly adding new strategies to analyze and monitor authenticity of agri-food products. The PCR-based methods proved to be reliable, fast and sensitive to detect rice adulteration and the use of emergent technologies such as ddPCR and KASP show high potential due to their specificity and speed.

#### 3.2.2. Post-PCR Detection Strategies (HRM, Bar-HRM)

High Resolution melting (HRM) is a post-PCR method that does not require separation or post-processing of the samples [117]. The method measures the rate of double strand DNA dissociation when raising the temperature to obtain single-stranded DNA [118]. The amplicons are distinguished based on their melting profiles [117]. HRM was already used in rice to perform rapid screening of rice mutants, being able to detect single nucleotide mutations [117]. This method was also used to uncover adulteration in Basmati rice samples, proving to be a highly sensitive tool able to detect a ratio of 1:100 of non-Basmati contamination in Basmati rice [59].

A method combining HRM with DNA barcoding, known as Bar-HRM [119], has shown to be successful for quantitative determination of adulterants in agri-food products [119,120,121] although it was still not explored in rice. This methodology uses sequences derived from barcoding markers, to design specific primers that allow the amplified region to be used in post-PCR HRM [119]. 

Bar-HRM allows quantitative analysis, in opposition to DNA barcoding, and it increases the resolving power of the conventional melting-curve analysis [120]. 

#### 3.2.3. Isothermal Amplification-Based Techniques

Isothermal amplification-based techniques are based on the exponential amplification of a specific nucleic acid region at a constant temperature, usually 60–65 °C, [47,122]. These methods may represent a promising alternative to PCR amplification, only requiring a thermoblock (the thermocycler is not needed) and a polymerase with high strand displacement activity in addition to replication activity. One of these methods, Loop-mediated amplification (LAMP), was applied to detect transgenic events in rice samples. By combining different primers recognizing multiple sequences of a target region, and adding an additional pair of “loop primers”, the LAMP-method allows the synthesis of large amounts of DNA in a short time. The products of the amplification can be detected with DNA-binding dyes or colorimetric indicators for naked-eye visual detection [122,123]. Recently, LAMP-based technology was used to develop a portable colorimetric assay kit (NIPPON GENE CO.) (https://nippongene-analysis.com/en/rice, accessed on 15 November 21) for rice Koshihikari cultivar discrimination. DNA detection of the Koshihikari genotype can be performed in both raw and cooked rice, representing an easy, fast and very specific method. The application of the method to discriminate other varieties of commercial interest remains to be explored, but the main disadvantage is that to cover different accessions of the same species, one needs to use multiple degenerated primers, which can reduce the specificity of the amplification. 

### 3.3. Progress in DNA-Based Methods for Rice Authentication

**Figure 4:**
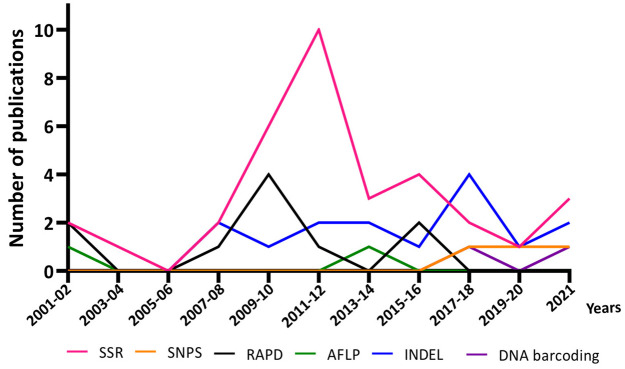
Number of publications per biennium, in the last 20 years, focusing on rice authentication through DNA-based methods. The data were obtained by searching in the SCOPUS database (www.scopus.com; accessed on 18 November 2021) articles published in 2001–2021 using the following terms: (Rice) AND (Authentication), (Rice) AND (Fraud), (Rice) AND (Molecular markers), (Rice) AND (Adulteration) and (Identification) AND (Rice) AND (Varieties). 2001–02: [67,83,87,127]; 2003–04: [128]; 2007–08: [57,60,129,130,131]; 2009–10: [54,78,130,132,133,134,135,136]; 2011–12: [59,86,116,123,137,138,139,140,141,142]; 2013–14: [55,58,81,143,144,145]; 2015–16: [89,111,146,147,148,149]; 2017–18: [53,103,108,150,151,152,153]; 2019–20: [52,154,155]; 2021: [51,104,112,114,156,157].

DNA-based methods provide a valuable and inexpensive tool for rice authentication aiming to tackle fraud and adulteration, with potential to be easily standardized worldwide. To have a general overview of the different strategies used in this context, we have explored the SCOPUS database over the last twenty years, using keywords to search for literature on rice fingerprinting and adulteration (Figure 4). In this search, SSRs showed up as the most used strategy in the last 20 years, although RAPDs were still prevalent between 2007 and 2015. The reduced use of RAPDs in recent times was expected given the disadvantages referred to in point 5.1.2.

Interestingly, only two articles were published using SNPs for rice authentication [52,114] in the last 3 years. This highlights the insufficient research in exploring this resource to tackle rice fraud and adulteration (Figure 4). With the current resequencing efforts, it is expected that the use of SNPs will rise due to the above-discussed advantages they show over other molecular markers. In fact, SNP strategies are already being implemented for authentication in other cereal species [124,125,126].

The identification of polymorphisms generated by insertion or deletion (InDel) of small sequences in target genes has also been used in the identification of varieties, particularly for aromatic/basmati rice (Figure 4) [58,114]. Through the valuable recent tools of next-generation sequencing, which provide high-speed and low cost, it was possible to find that non-aromatic rice contains a protein, known as BAD2, that is absent in basmati rice [57]. The missing protein in basmati rice is the result of a deletion of eight base pairs in this locus, as revealed by three SNPs in the *BAD2* gene [57,58,60]. This polymorphism allowed the detection and quantification of basmati adulteration.

The polymorphic diversity of rice genomes and the high reproducibility of the classical molecular markers have been only grasped as target for rice authentication strategies. SNPs and SSRs, in particular, show high potential for scaling-up in automated platforms.

**Table 1:** DNA-based techniques used for cultivar identification, mentioned in this review.

Genotyping Methods	Type of Molecular Marker	Cultivars	References
Probe/enzyme	RFLP	*indica* vs. *japonica*	[63]
PCR	PCR-RFLP	Aromatic varieties	[64]
	RAPD	Aromatic varieties	[67]
		48 rice lines	[149]
	RAPD/SCAR	Jasmine rice	[54]
	AFLP	6 glutinous rice varieties	[89]
	SSR	Basmati vs. NB	[80]
		Indian rice hybrids	[137]
		Basmati vs. advanced lines	[142]
		60 varieties	[155]
	EST-SSR	Fine-grain varieties	[86]
	ISSR	*japonica* vs. *indica*	[88]
	ISSR, SSR	Basmati vs. EB vs. NB	[87]
	SSR, ISSR, RAPD	30 indica varieties	[148]
	InDel	*japonica* vs. *indica*	[129]
Multiplex PCR	SSR	Basmati vs. NB	[57] *^,1^
		Long and medium-grain rice varieties	[53]
		13 rice cultivars	[83]
		Variety: Samba Mahsuri	[84]
	STS	130 varieties	[100]
Duplex PCR	InDel	Basmati vs. NB	[58] *
	SSR	Basmati vs. NB	[81] *^,1^
Duplex Digital Droplet PCR	InDel	Basmati vs. NB	[58] *
TaqMan PCR	SNP and InDel	Basmati vs. NB	[60] *
KASP	SNP	*indica* vs. *japonica*	[98]
	SNP and InDel	Basmati varieties; Basmati vs. NB	[114]
Fluidigm (PCR in Dynamic array IFCs)	SNP	*indica* vs. *japonica*	[101]
HRM	SSR and InDel	Basmati vs. NB	[59] *

* Quantification of adulteration. ^1^ capillary electrophoresis. NB—Non-basmati; EB—Evolved basmati. IFC—integrated fluidic circuit.

## 4. Conclusions

Authentication of the numerous rice varieties being cultivated and commercialized worldwide is imperative to avoid adulteration and fraud in the rice food-chain. DNA based techniques have proven to be a valuable tool, not only to detect adulteration and fraud, but also to accurately and robustly discriminate rice species and varieties (Table 1).

Nowadays, the increase of whole-genome rice data, boosted by the 3K project and led by the advances in sequencing technologies and bioinformatic tools, opened the door for the identification of an exponential number of new DNA-based markers. This will definitely contribute to the development of new methodologies for rice authenticity analysis. The fraud detection methods should focus on simplicity, reliability, low-cost and high-throughput to monitor safety and quality along the rice value-chain.

All the DNA-based methods, however, can only target DNA features and thus, all phenotypic traits, including physicochemical properties and metabolite composition or stability, must be assessed through other strategies. Additionally, the environmental impact on gene expression and grain quality is not something traceable by DNA-based methods. For instance, the same genotype cultivated in different edaphoclimatic or agro-management conditions may yield rice with diverse properties. Different detection strategies must therefore be applied when searching for general fraud problems including the certification of geographical origin or cultivation method.
